# Examining the prevalence and type of technology-use in people with Down syndrome: Perspectives from parents and caregivers

**DOI:** 10.1177/17446295231176121

**Published:** 2023-05-18

**Authors:** Su Morris, Emily K. Farran, Katie A. Gilligan-Lee

**Affiliations:** School of Psychology, 3660 University of Surrey, Guildford, UK; School of Psychology, 1948 University of Sussex, Falmer, UK; School of Psychology, 3660 University of Surrey, Guildford, UK; Centre for Educational Neuroscience, 4894 Birkbeck University of London, London, UK; School of Psychology, 3660 University of Surrey, Guildford, UK; Centre for Educational Neuroscience, 4894 Birkbeck University of London, London, UK; School of Psychology, 8797 University College Dublin, Dublin, Ireland

**Keywords:** Down syndrome, gaming, questionnaire, social media, technology-use

## Abstract

Familiarity with technology has become a requirement for independent living, however there is limited information on technology access and use for people with Down syndrome (DS). The aim of this study is to describe technology, gaming, and social-media use in people with DS. Parents/caregivers (*N* = 220) of individuals with DS aged 5–35 years (49% female) completed an online questionnaire. They felt that technology and social media use, and to a lesser extent gaming, played an important role in their son/daughter’s life. However, many had concerns about their son/daughter’s safety online, and identified challenges they faced with using technology, such as using a mouse and speech recognition. We also found substantial parental interest in learning more about technology-use in DS, particularly the impact of using social media. This paper summarises important details about technology-use in people with DS, providing foundational information for the design of effective technology-based activities and support.

## Introduction

Since the dawn of the digital revolution in the 1980’s, life without technology has become increasingly inconceivable. For young people, technology in the form of televisions, computers, games consoles, and mobile phones, now plays a key role in their lives, from entertainment, to socialising, to sharing information (Ofcom, 2021; Strasburger & Hogan, 2013). However, not all population groups have equitable access to technology; a fact that has resulted in a “digital divide” with differing access to, and usage of technological devices across populations (Dewan & Riggins, 2005; Scholz et al., 2017). One such population is people with Down syndrome (DS), the most prevalent genetic syndrome which occurs in 25 out of 10,000 births in the UK; a level which differs internationally with varying incidence of elective terminations (de Graaf et al., 2021; Public Health England, 2020). DS is characterised by mild to severe intellectual difficulties, poor verbal abilities relative to visuo-spatial abilities, and some additional physical health problems (Silverman, 2007; Yang et al., 2014). Despite the known importance of technology in typical development, there is little empirical evidence on the role that technology plays in the lives of those with DS and their families. This study provides the most extensive insight into technology-use in individuals with DS to date and highlights the potential of future technology-based interventions for people with DS. Given that familiarity with technology has become a requirement for independent living, it is imperative that technology is made accessible to all.

### Technology-use in typically developing young people

Technology-use is prevalent in young people in the UK with most (96%) households having access to the internet, and children (5-15 years) listing smart TVs, tablets, mobile phones, computers (desktops, laptops, and netbooks) and games consoles as their preferred devices (Ofcom, 2021). Similar patterns are found within the EU. The EU Kids Online project found that between 65% (France) and 89% (Lithuania) of 9-16-year-olds use a smart phone daily with children spending between 2 hours 14 minutes (Switzerland) and 3 hours 39 minutes (Norway) online every day. The most common online activities were watching videos, listening to music, communicating, using social media and playing online games (Smahel et al., 2020).

The uses of technology are wide ranging. Technology supports educational learning and many cognitive/behavioural interventions rely on technology to deliver training (e.g., Gilligan et al., 2020; Klingberg et al., 2005; Outhwaite et al., 2017; Uttal et al., 2013). Beyond education, technology facilitates leisure-time activities including gaming and social media. Young people (5 to 15 years) in the UK play video games using a console, phone or tablet for on average 1 hour 21 minutes per day (Ofcom, 2021), while 87% of young people (12-15 years) use social media, with Facebook, Instagram, and Snapchat the most popular platforms (Ofcom, 2021).

Research has highlighted several benefits of computer-based gaming, including improving processing speed without diminishing accuracy (Dye et al., 2009) and improving deployment of visual attention (Bavelier & Davidson, 2013). Gaming can positively impact social development, with positive associations reported between prosocial gaming and prosocial behaviours in the physical world (Gentile et al., 2009). Conversely, violent gameplay has been associated with lower helping behaviour and lower empathy in young adolescents (Gentile et al., 2009), and increased conduct disorder at 15 years (Etchells et al., 2016). A similar pattern of pros and cons exists for social media use. Social media use has a positive effect on the development of prosocial behaviours, positive relationships with peers, widening social contacts, and engaging in social activities in individuals with limited access to real-world social interaction (Spies Shapiro & Margolin, 2014). However, social media-users often have to deal with negative feedback, pressure to share personal details, and unhealthy comparisons about physical appearance and lifestyle (Spies Shapiro & Margolin, 2014; Tiggemann & Slater, 2014). More detailed reviews on the advantages and challenges of social media and gaming have been provided previously (Brilliant, Nouchi, & Kawashima, 2019; Gottschalk, 2019; Punukollu & Marques, 2019).

The challenges of technology use also evoke parental concern. Parents worry about children’s over-reliance on technology, reductions in real-world communication, online bullying and accessibility to unsuitable content, among others (Nikolopoulou, 2020, Ofcom, 2021). Findings from the EU Kids Online project that show an increase in parental mediation of children’s technology-use since 2010, however this includes less restrictive practices and a greater focus on guiding children in internet use (Kalmus et al., 2022).

### Technology-use in atypically developing populations

Although the current study focuses specifically on people with DS, the wider literature on people with intellectual and developmental disabilities (IDD) provides important insights for our work. There is evidence that technology and internet use is lower in people with IDD. Alfredsson Ågren, Kjellberg, and Hemmingsson (2020) reported significantly lower access to internet-enabled devices and use of internet activities in adolescents (13-20 years) with an intellectual disability compared to their typically developing peers. They suggested that this digital divide may be attributable to the cognitive demands of the internet, a hypothesis supported by Ramsten et al., (2020) who found that technology-use was limited by difficulties with reading and other challenges associated with intellectual disability. Beyond frequency of use, there is also evidence that people with IDD access and use technology in different ways to those with typical development, and many have developed strategies to support their technology use, e.g., getting support from others, using picture-based strategies like using icons, or using word-based strategies like hand written notes containing passwords or tips for reaching higher levels of computer games (Alfredsson Ågren, Kjellberg, and Hemmingsson, 2020b).

However, technology can also offer support and independence to those with IDD, when applied appropriately. A study which surveyed family members of individuals with IDD found that technology was used for mobility, hearing and vision support, communication, and supporting independent living such as using schedulers and alert buttons (Palmer et al., 2012). There is also evidence that technology is an effective means of teaching skills. For children with DS (aged 6-8 years) greater gains in counting were reported through multimedia learning than using pen-and-paper, highlighting the potential of technology in delivering educational interventions for this group (Ortega-Tudela & Gómez-Ariza, 2006).

Social media specifically also has the potential to support social connections in people with IDD. Adolescents with IDD report using technology for virtual social interactions (social media and texting), and describe its potential to increase their independence (Ramsten et al., 2020). Similarly, Shpigelman and Gill (2014) found that people with IDD use Facebook as often as typically developing peers, despite challenges with privacy settings and literacy demands. Interviews revealed that Facebook-use led to increases in bonding social capital and psychological well-being by improving participants’ online visibility, popularity and sense of belonging (Shpigelman, 2018). In another study the use of mobile technology apps amongst those with IDD was positively associated with feelings of social inclusion with family, friends, and work/volunteering (Martin et al., 2021). To this end, there is some evidence that interventions that provide social media training and instruction on adaptive technology may lead to improved social participation, independence and literacy, although the sample size in this study was very small, (N=9) (Raghavendra et al., 2018).

Despite its potential to enhance independent living and offer intervention there is limited research into technology-use in people with DS. Given the specific cognitive profile of individuals with DS, understanding how this population accesses technology, will allow tailoring of technology-based tools for this group. A US-based study of 107 caregivers across nine countries found that individuals with DS aged 10 to 25 years frequently used tablets (88%), mobile phones (88%), and computers (75%), and technology was used for leisure by 93% of the sample (Fritz, 2017). Most caregivers felt that the use of technology played a large to moderate role in their son/daughter’s everyday life, and half reported placing restrictions on their son/daughter’s technology-use such as limiting time or type of activity. Interestingly, the author noted some differences in technology-use between countries such as a higher level of parental restrictions on devices in countries outside the US (Fritz, 2017). Further detailed research is needed to fully understand the type and prevalence of technology-use, as well as barriers to its use, in children, adolescents, and adults with DS. Given the rapid rise in use of digital technologies, there is a need for up-to-date country-specific figures that incorporate new technological advancements.

Finally, it is noteworthy that differing perspectives on technology and social media use in people with IDD can be taken from parents/caregivers and from individuals with IDD themselves. Heitplatz, Bühler, and Hastall (2022) found that caregivers in outpatient facilities and residential homes had more negative attitudes to internet-use for people with IDD owing to technology access issues, accessing inappropriate information and vulnerability to data abuse and cyber bullying. They did acknowledge that smart phones provided autonomy and new avenues for communication in those with poor literacy, e.g., using emojis. In the same study, individuals with IDD had more positive attitudes, they enjoyed easy access to the internet and to social communication through social media platforms like WhatsApp and Facebook. They also noted that access to the internet provided a sense of security, of being available to others and feeling like they belong, despite the limitations, e.g., remembering pins, financial cost, requiring support. Similarly, Chiner et al., (2017) found significantly higher estimates of the frequency (computers and tablets only) and type of technology-use (computers only) reported by caregivers than individuals with IDD themselves, with no significant differences relating to other devices. Importantly, a high proportion of caregivers did not know about problem incidents when online (39%) or whether their son/daughter had engaged in inappropriate behaviour (49%). Of those who were aware, there were some discrepancies between caregivers and IDD in reports of internet risks (with a lower proportion of caregivers reporting incidents than individuals with IDD) and engaging in inappropriate online behaviour (with a higher proportion of caregivers reporting incidents than individuals with IDD).

By comparison, our study design included a wide age range (including children as young as 5 years) because we know that technology-use spans this age range in typical development. As it would be difficult to elicit self-report from individuals across our age range, the focus was on parent/caregiver perspectives only. However, we acknowledge that different and insightful insights would also be found by surveying individuals with DS directly.

### The impact of COVID-19 on technology use

This study was conducted between June and October 2020, i.e., after the UK population had experienced strict COVID-19 related lockdowns, and at a time when the country remained subject to social distancing restrictions. The unprecedented social climate at the time of this study is an important factor in understanding our findings. Of particular relevance, there was a sudden reduction in social interaction and a related increased need for people to quickly adapt to online communication and the use of digital technologies. Previous findings from McCausland et al., (2021) show that both access to, and use of technology increased post lockdown in people with IDD (including people with DS). Indeed, Navas et al., (2021) found that using technology to contact others was a key support that made people with IDD feel good during lockdown (30% of 582 people with IDD reported this). From the UK, Caton et al., (2022) found that across 571 individuals with IDD, over 70% had access to the internet during COVID-19. For adolescents and young adults (16-34 years) technology was used most for communicating with family/friends (76%), streaming TV and film (66%), using social media (79%), doing online activities with others (66%), and online shopping (44%). For the oldest group (55+ years), the number of participants completing each of these activities was substantially reduced (42%, 30%, 26%, 50% and 13% respectively). Thus, showing not only the breath of activities for which technology plays a role in the lives of those with IDD, but the influence of age in moderating these patterns. Despite its benefits, Chadwick et al. (2022) reviewed papers on technology-use in those with IDD during COVID-19 and found that technology-use had clear challenges including dependency on support for internet access, concerns over online security, limitations due to prior digital literacy, and financial costs. In short, previous studies show that COVID-19 lockdowns led to changes in technology-use patterns, however, the majority of this work is based on adult populations and includes those with varying intellectual disabilities. To best propose tech-based interventions, more nuanced findings are required.

### Current study

The overarching purpose of this study is to generate insights about the prevalence and type of technology-use in people with DS . This will lead to two outcomes. First, this study will give parents and carers of people with DS access to information about technology-use in the wider DS community. Second, the findings will have broader applications for researchers and professionals who work with individuals with DS, for the selection of appropriate technology-based tools for training and intervention designed for individuals with DS. This study provides the most detailed data on technology-use in people with DS to date, including descriptive and associational analyses. This will allow us to compare the use of different technological devices, the different activities technology is commonly used for, and engagement with gaming and social media. It will also provide insight into the extent to which people with DS can access technology independently, any necessary hardware and software adaptations they require, and parental concerns associated with technology-use.

The study has three broad research aims:

1) To identify patterns of technology, gaming and social media use in individuals with DS.
2) To establish associations between parental characteristics (e.g., parental confidence and concern about using technology) and technology-use in individuals with DS.
3) To determine the impact of individual differences (e.g., age, eye-sight) on technology, gaming and social media use in individuals with DS.

## Method

### Participants

Participants were parents/caregivers with a son or daughter with DS between the ages of 5 years and 35 years, from the UK or Ireland. An upper age limit of 35 years was selected as people with DS can be diagnosed with early-onset dementia typically from 40 years (Head et al., 2012), which may have an impact on their patterns of technology-use. A lower limit of 5 years was chosen to reflect the fact that technology-use is known to be prevalent in this age group in typical development, e.g., 57% of 5- to 7-year-olds own their own tablet (Ofcom, 2021). We were interested to investigate whether similar patterns would be evident in children with DS. Furthermore, children in the UK start school by 5 years, thus benchmarks of technology-use at this age are key to the development of effective school-based learning interventions. Our recruitment target was 178 participants. This was determined using GPower for the largest proposed analysis in our analysis plan, an ANOVA with 4 groups (power 0.8; alpha 0.05; effect size of 0.25). More details can be found in our pre-registration (https://osf.io/4ku2c/). The final number of participants who completed the questionnaire was 220. A summary of demographic information for the sample can be viewed in Table 1. Please note that all demographic information relates to the individuals with DS about whom parents/caregivers completed the questionnaire.

**Table 1.** Demographic information.

Variable	Categories	
Mean age	Years; months	15;8
		Range: 5;0 - 34;7
Gender (%)	Male	112 (50.9%)
	Female	108 (49.1%)
Country of Residence (%)	United Kingdom	129 (58.6%)
	Ireland	89 (40.5%)
	Unanswered	2 (0.9%)
Additional diagnoses^a,b^	None	151 (68.6%)
	Motor	9 (4.1%)
	Health	34 (15.5%)
	Senses	19 (8.6%)
	Behavioural	25 (11.4%)
Requires glasses (%)	Yes	167 (75.9%)
	No	53 (24.1%)
Additional visual difficulties (%)^b^	Nystagmus	19 (8.6%)
	Keratoconus	2 (0.9%)
	Strabismus	49 (22.3%)
	None	153 (69.5%)
Quality of vision with glasses (%)	Better than average	27 (12.4%)
	Average	173 (79.4%)
	Poor	12 (5.5%)
	Very poor	6 (2.8%)
	Unanswered	2 (0.9%)

^a^These were broad categories of additional diagnoses (see Supplementary Information for further details).

^b^Some participants had more than one difficulty, so the percentages total more than 100%.

### Questionnaire

A novel questionnaire, the Technology, Gaming and Social-media Survey (TOGSS) was designed for use in this study. This can be found on the Open Science Framework (OSF) (https://osf.io/4ku2c/). It took an average of 20 minutes to complete and was delivered online using the Qualtrics platform. The questionnaire included four sections: 1) participant’s general use of technology; 2) prevalence and type of gaming; 3) prevalence and type of social media use; 4) engagement with non-technology activities. There were two initial stages of questionnaire development before the study was launched. We drafted our questionnaire items based on previous similar questionnaires (e.g., Fritz, 2017). The choice of what social media platforms to use was based on previous studies of technology and social-media use in typically developing groups (Ofcom, 2019). We next provided this draft questionnaire to three parents of individuals with DS and asked them to review it for suitability and relevance to people with DS. We had informal interviews with these parents to gain feedback. The questionnaire was modified based on parental suggestions. Next, we asked a sample of ten neurotypical adults to complete the questionnaire which allowed us to pilot the logic and understandability of the questions and instructions. A questionnaire was used as the mode of data collection in this study due to: 1) practical limitations of completing interviews during the covid-19 pandemic, and the requirement for participation time to be short due to the additional time pressures on parents/caregivers during this period; 2) a desire to maximise sample size and consequently the generalisability of the findings, hence an online questionnaire was deemed a more efficient way to collect data from a larger sample.

### Procedure

The study received favourable ethical approval from the University of Surrey. Participants were recruited online via social media using Facebook and Twitter. Information was also sent to DS support groups via email, Facebook, and Twitter. Following the completion of a consent form, respondents answered a short demographic questionnaire, followed by the TOGSS. All data were collected between June and October 2020.

### Statistical analyses

The analysis plan was pre-registered and can be viewed on the OSF alongside an anonymous data file for this study (https://osf.io/4ku2c/). The analyses were largely undertaken using Jamovi, and the R code can be viewed on the OSF. A number of continuous scale variables were created. Two variables were calculated from the estimated number of minutes spent per day on different activities: Overall technology-use (sum of time spent on different devices per day), and overall non-technology (sum of time spent on different non-technology activities per day). Five continuous variables were also created from responses to forced-choice statements (Table 2). The response options for each of these scales were ‘definitely agree’, ‘slightly agree’, ‘slightly disagree’, and ‘definitely disagree’ and items were reverse-coded where required. Responses were scored from 1 to 4 across the response options for each statement, with a higher overall score denoting greater difficulty, a higher level of concern, or a higher level of confidence, depending on the variable. Cronbach’s alpha scores indicate a good to acceptable level of reliability for all scales (Field, 2013) (see Table 2). Descriptive statistics were used to summarise data from categorical questionnaire items. To investigate individual differences related to technology-use, correlations, chi-squared analyses, and analysis of variance (ANOVAs) were used. There were some questions with a free-text response, including difficulties encountered, and monitoring activities. These responses were analysed using thematic analysis by two researchers. Inter-rater reliability was 100%. Note some analysis, not central to the main research questions, can be found as supplementary materials on the OSF due to word constraints.

**Table 2.** Variables created from responses to forced-choice statements.

Scale	Number of statements	Range of scores	Reliability (Cronbach’s α)	Higher score shows greater…
User-difficulty scale	7	7 to 28	.735	Difficulty
Parental confidence (use)	5	5 to 20	.722	Confidence
Parental confidence (support)	3	3 to 12	.822	Confidence
Parental concerns about social media	6	6 to 24	.746	Concern
Parental information gap	5	5 to 20	.885	Information gap

Note: For further information about the statements creating these variables, see section 3.1.4.

## Results

### Patterns of technology-use across the sample

#### Different types of technology, gaming and social-media use

**Figure 1.**
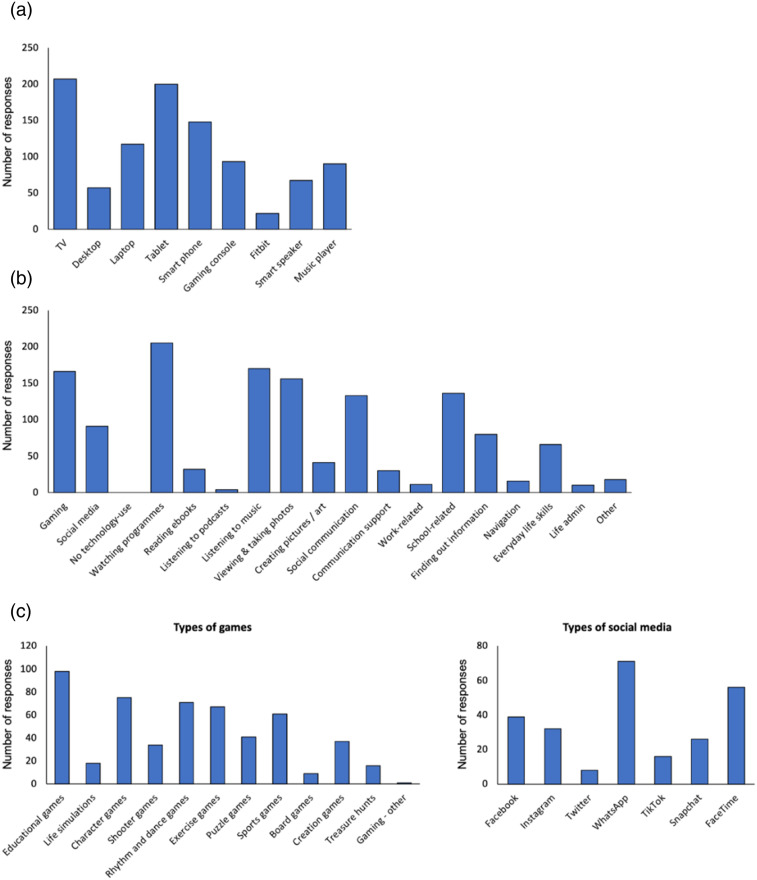
Different activities undertaken by people with Down syndrome using technology. (a) Types of device used. (b) Activities that technological devices are used for. (c) Types of games and different social media platforms used by people with Down syndrome.

Parents/caregivers were asked to report which devices and activities their son/daughters used. This question did not specify a timescale or frequency of use, and therefore provides a general picture of devices, activities, and platforms used by people with Down syndrome. Across the whole sample, the most frequently-used devices were televisions and tablets, followed by smart phones and laptops, and to a lesser extent, gaming consoles and music players (Figure 1(a)). This is also reflected in the activities most often undertaken using technology: watching programmes, listening to music, gaming, and viewing and taking photos (Figure 1(b)). The most frequently-cited activity in the free-text ‘other’ option was creating music, videos, and writing (4.1% of participants). The results show that while 75.5% of participants participated in gaming, only 41.4% used social media. However, when children aged 12 years and under were excluded, 65.3% of the sample used social media. Educational games were most popular, followed by character games, rhythm and dance games, exercise games, and sports games (Figure 1(c)). The two most popular social media platforms were WhatsApp and FaceTime, and the most frequently-cited ‘other’ option was Zoom (3.6% of the sample).

Due to the wide age range of our sample, we additionally examined how these patterns varied according to age group (children: 5-12 years; teenagers: 13-17 years; adults: 18 years plus). A Chi-squared analysis (3 x 5) was carried out to compare use of technological devices across age groups (children, adolescents, adults). These revealed that children were significantly less likely to use a laptop/desktop computer, *X*^2^ (1, *N*= 220) = 24.36, *p* < .001, a tablet device, *X*^2^ (1, *N*= 220) = 16.18, *p* < .001, a smart phone, *X*^2^ (1, *N*= 220) = 34.11, *p* < .001, or a gaming console, *X*^2^ (1, *N*= 220) = 26.80, *p* < .001, than adolescents and adults. We merged the data for smart speakers, Fitbit, and music players and found that a significantly greater proportion of adults used these devices compared with children and adolescents, *X*^2^ (1, *N*= 220) = 24.25, *p* < .001.

A similar Chi-squared analysis (3 x 5) was used to compare whether technology-based activities varied across age groups (children, adolescents, adults). There was no difference in the proportions of individuals across different age groups, who used technology for: leisure, i.e., watching programmes, listening to podcasts, reading ebooks, or listening to music, *X*^2^ (1, *N*= 220) = 0.20, *p* =.904; creativity, i.e., viewing and taking photos, or creating pictures/art, *X*^2^ (1, *N*= 220) = 2.91, *p* =.233; or independent living, i.e., navigation, sourcing information, school-related activities, work-related activities, online life administration, *X*^2^ (1, *N*= 220) = 3.59, *p* =.166. There was a higher proportion of adults and adolescents gaming compared with children, *X*^2^ (1, *N*= 220) = 6.37, *p* = .041. There was also a much higher proportion of adults, and a lower proportion of children, using technology for communication, *X*^2^ (1, *N*= 220) = 27.63, *p* <.001.

#### Importance of technology

**Figure 2.**
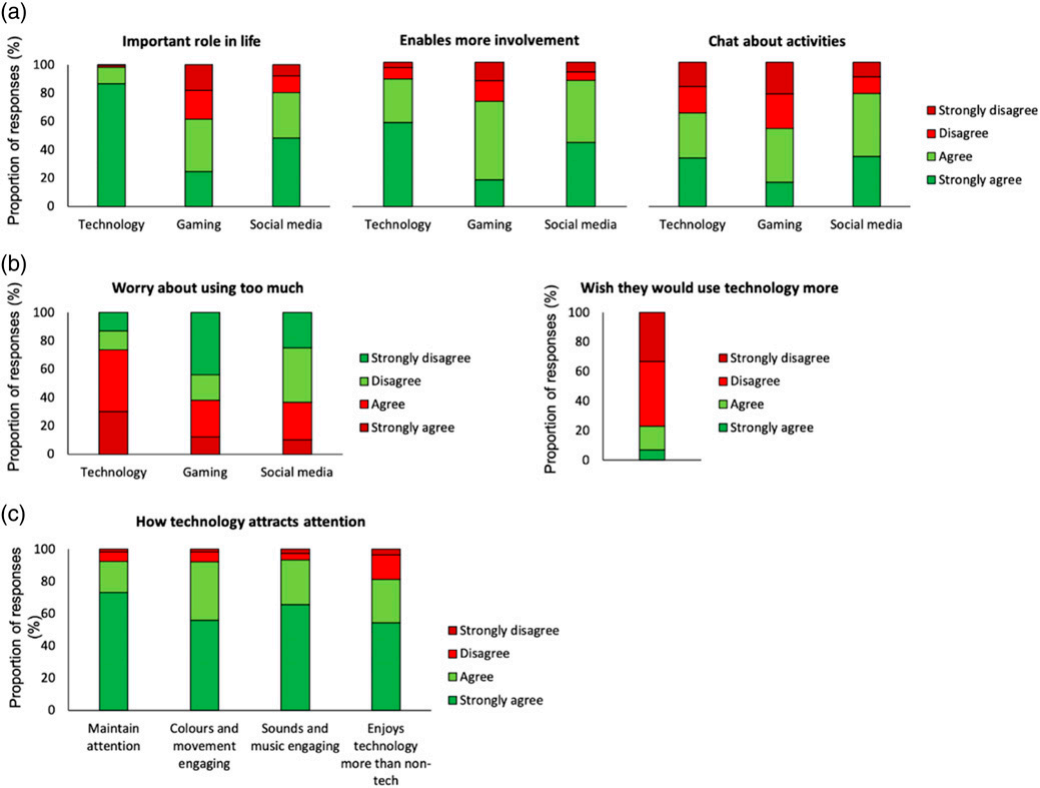
Proportion of parent/caregiver responses about their son/daughter’s engagement with technology. (a) The importance of technology, gaming, and social media to people with Down syndrome. (b) Parent/caregiver concerns about level of technology-use. (c) Features of technology activities that may maintain attention and engagement.

The majority of respondents reported that technology (98.2%) and social media (79.5%) played an important role in their son/daughter’s life (Figure 2(a)). Fewer respondents stated that gaming played an important role (61.6%), but this was higher for teenagers than for children or for adults, χ^2^(6, N=164) = 14.20, *p* = .027. Parents/caregivers generally reported that technology, gaming and social media enabled their son/daughter to feel more involved, and over half reported they could chat about the activities with their son/daughter (technology: 65.0%; gaming: 54.0%; social media: 78.2%). However, well over half of parents/caregivers reported that they believed their son/daughter used technology too much (73.5%), particularly in the case of teenagers compared with children and to adults, χ^2^(6, N=219) = 17.40, *p* = .008. In the specific areas of gaming and social media, levels were lower (37.8% and 36.6% respectively; Figure 2(b)), although again, for gaming, this was more of a concern for parents/caregivers of teenagers than children or adults, χ^2^(6, N=164) = 20.50, *p* = .002. Conversely, almost a quarter of caregivers (23.0%) stated a desire that their son/daughter would use technology more than they currently do, with no significant differences across age group.

From the perspective of accessibility and engagement, most respondents (83.5%) said that their son/daughter preferred using touchscreen technology over a mouse or trackpad, although this proportion was lower for adults than for children or for teenagers, χ^2^(6, N=218) = 45.10, *p* < .001. Parents/caregivers reported that their son/daughter could maintain attention for longer on an activity based on technology than a pen-and-paper activity (92.3%), and that both visual (92.2%) and auditory (93.2%) effects made technology-based activities more engaging (Figure 2(c)). Only 18.7% of parents/caregivers stated that their son/daughter did not enjoy technology-based activities more than those not involving technology.

#### Concerns and difficulties with technology-use

**Figure 3.**
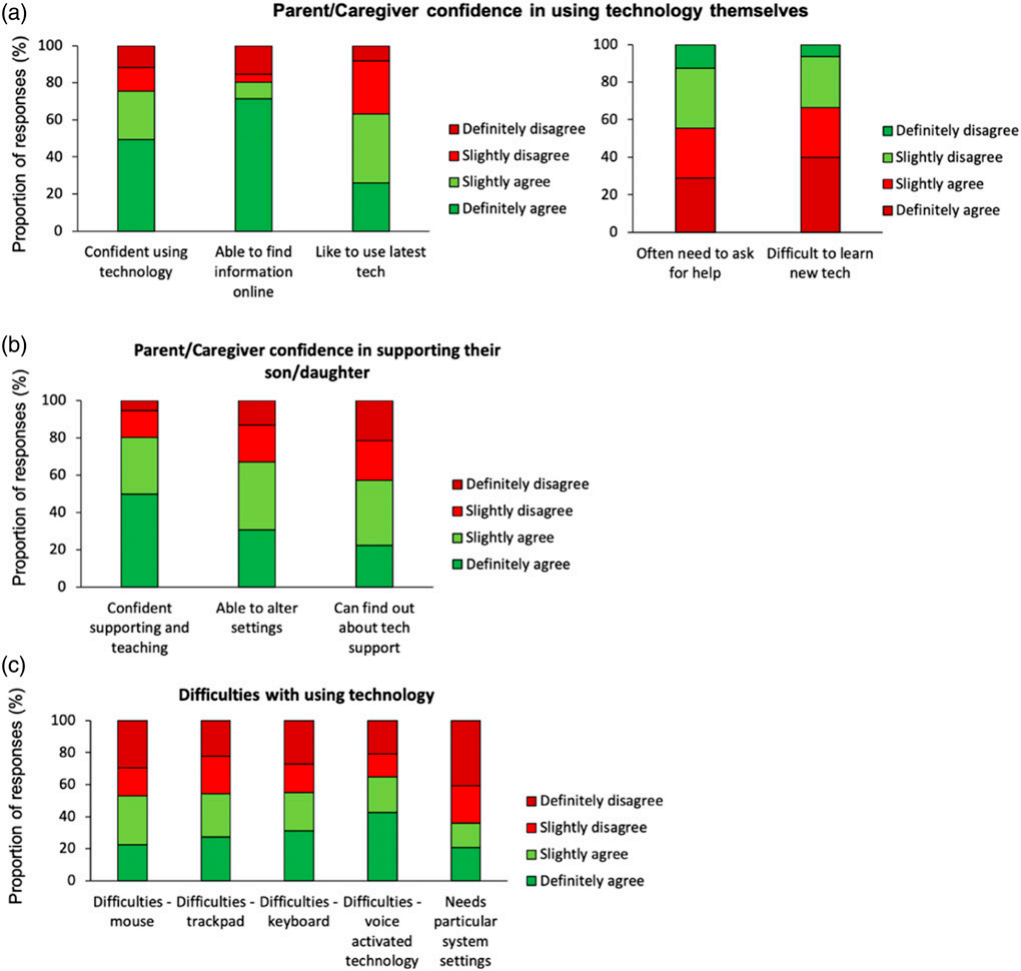
(a) Parent/caregiver confidence in using technology themselves. (b) Parent/caregiver confidence in supporting or teaching their son/daughter to use technology. (c) Difficulties people with Down syndrome have with using technology.

Most parents/caregivers said that they were confident using technology themselves (75.5%) and were able to find information online (80.5%). However, over half responded that they often needed to ask for help (55.5%) and that it was difficult to use new technology (66.4%) (Figure 3(a)). Most parents/caregivers were confident in supporting their son/daughter with their technology-use (80.4%) and knew how to alter settings to improve accessibility (67.1%), while just over half knew how to find out about new technology to support their son/daughter (57.3%) (Figure 3(b)). In terms of challenges encountered by individuals with DS when using technology, just over half had difficulties with using a mouse (53.0%), trackpad (54.5%), keyboard (55.1%) and voice-activated technology (64.9%), and about a third (36.1%) required specific system settings (Figure 3(c)). Only 20.5% could operate technology independently, and only 14.2% found it easy to learn how to use technology.

**Figure 4.**
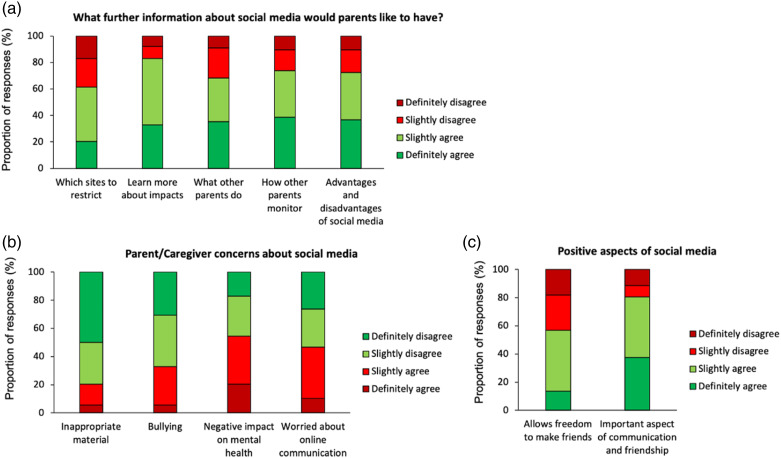
Parent/Caregiver concerns about social media. (a) Further information about social media that parents/caregivers would like to have. (b) Parent/caregiver concerns about social media. (c) Positive aspects of social media.

Parents/Caregivers of individuals who used social media (mainly adolescents and adults) were particularly keen to understand more about the impact social media might have on their son/daughter (83.0%) and the advantages and disadvantages of social media use (72.4%). There was also a strong interest in finding out whether other parents/caregivers allow their son/daughter to use social media (68.2%) and how they monitor their son/daughter’s activities (73.9%) (Figure 4(a)). The most frequently-held parent/caregiver concerns relating to social media was that it might have a negative impact on their son/daughter’s mental health (54.5%) and that they worry more about online communication than in-person communication (46.6%) (Figure 4(b)). There were fewer concerns about their son/daughter accessing inappropriate material (20.5%) or online bullying (33.0%) but the percentages still indicate that these are important concerns to parents/caregivers. Over half (56.8%) of parents/caregivers felt that social media gives their son/daughter freedom to make friends and 80.7% felt that social media was an important aspect of communication and friendship for their son/daughter (Figure 4(c)).

In order to improve access to technology, 7.3% said that their son/daughter had some hardware adaptations, mainly colour-coded keys, large keys, small keys, special mouse, and touchscreen. 27.3% said their son/daughter used software to support their learning. Of these, the most frequently-used type of software support were reading and writing apps (76.7%) and multi-subject learning apps (50%). Only 6.7% said that their son/daughter used an app specifically to support mathematics.

**Figure 5.**
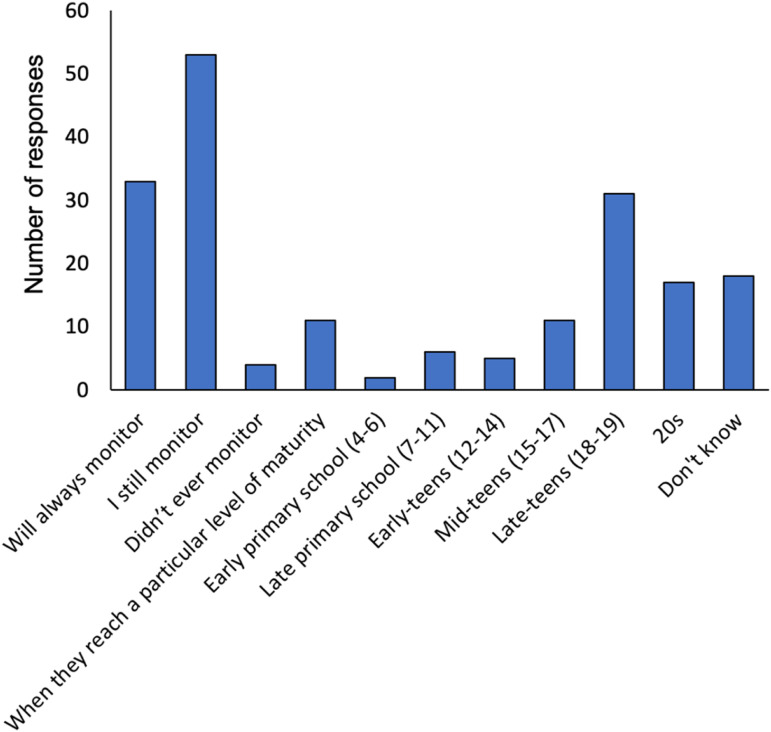
Categories of responses to the question about when caregivers would stop monitoring their son/daughter’s technology-use.

The free-text responses regarding the monitoring of their son/daughter’s technology-use, revealed that many caregivers would always monitor (17.3%) or that they are still currently monitoring (27.7%). Others stated they would stop at a particular age, most-often aged 18 years or older (66.7% of responses with a specific age), while 5.8% said that they would stop monitoring once their son/daughter reached a certain level of maturity (rather than a specific age) (Figure 5).

### Associations between parental characteristics and son/daughter’s technology-use

Pearson’s correlations (Table 3) show that higher parental concern about social media related to a lower level of social media-use by their son/daughter (*p* = .018) but there were no other significant associations with technology, gaming, or social media use. There was a significant association between greater caregiver concerns about social media and a greater desire for more information about technology (*p* < .001) and lower confidence in using technology (*p* = .034). There was also a high association between caregiver confidence in using technology and caregiver confidence in supporting their son/daughter (*p* < .001).

**Table 3.** Pearson’s correlations between use of technology, gaming, and social media, and parental concerns and parental confidence.

			1	2	3	4	5	6
1	Overall technology-use	Pearson’s r						
		*N*						
2	Overall gaming-use	Pearson’s r	.431 ***					
		*N*	135					
3	Overall social media-use	Pearson’s r	.289 **	-.090				
		*N*	85	57				
4	Parental concern social media	Pearson’s r	-.117	-.006	-.259 *			
		*N*	88	60	84			
5	Parental information gap	Pearson’s r	-.082	-.068	-.041	.415 ***		
		*N*	88	60	84	88		
6	Parental confidence (tech-use)	Pearson’s r	.033	-.041	-.058	-.226 *	-.209	
		*N*	220	135	85	88	88	
7	Parental confidence (support & teach)	Pearson’s r	.025	.117	-.049	-.186	-.276 **	.560 ***
		*N*	218	134	84	87	87	218

Note: * p < .05, ** p < .05, *** p < .001.

**Figure 6.**
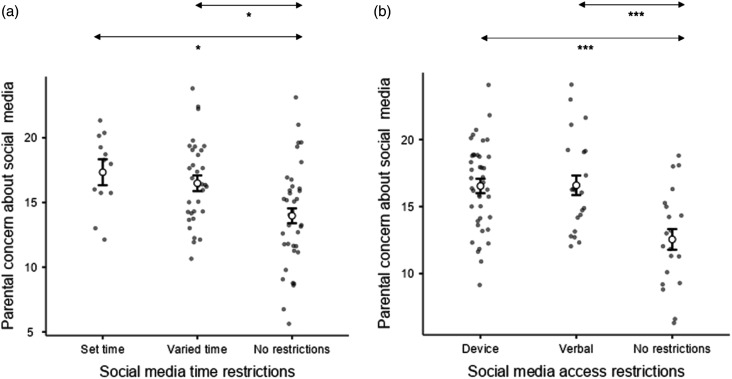
Estimated marginal means plots showing the group differences in parental concern about social media in the posthoc Tukey comparisons. (a) Parental concern grouped by social media time restrictions. (b) Parental concern grouped by social media access restrictions. * *p* < .05, ** *p* < .01, *** *p* < .001.

Two one-factor ANOVAs were carried out to examine whether the son/daughter’s gaming (DV1) and social media-use (DV2) varied according to parents’/caregivers’ own gaming or social media-use (IVs). This was measured by their level of agreement (4 levels) to a statement that they regularly play games or use social media. There was no significant difference in the son/daughter’s use of gaming (*p* = .317, η_p_^2^ = .026, BF_10_ = 0.200) or social media use (*p* = .471, η_p_^2^ = .031, BF_10_ = 0.282) depending on whether parents/caregivers regularly played games or used social media respectively. Further ANOVAs found that, surprisingly, the level of time restriction (IV: set amount of time, adhoc restriction, no restriction) from the parent/caregiver did not affect the amount of gaming (*p* = .357, η_p_^2^ = .016, BF_10_ = 0.208), or social media use, (*p* = .480*;* η_p_^2^ = .019, BF_10_ = 0.212). Similarly, access restrictions on social media use (IV: restrictions on device, son/daughter is aware of restrictions, no restrictions) did not affect the level of social media-use, (*p* = .931*;* η_p_^2^ = .002, BF_10_ = 0.122). It was not possible to compare restrictions across social media and gaming due to the small number of responses in some of the cells of the chi-squared analysis. ANOVAs revealed that those who set different levels of restrictions on social media (Time: IV1, and Access: IV2) had different levels of parent/caregiver concerns about social media (DV), Time: *F*(2,79) = 6.53, *p* = .002, η_p_^2^ = .142; Access: *F*(2,80) = 12.30, *p* < .001, η_p_^2^ = .235. Posthoc Tukey comparisons showed that those with no time restrictions had a lower level of parent/caregiver concern than both those with some time restrictions (*p* = .010, *d* = 0.721) and those who had a set amount of time allowed on social media (*p* = .013, d = 0.964) (Figure 6(a)). Similarly, those with no access restrictions had a lower level of concern than those with device restrictions (*p* < .001, *d* = 1.1603) and those where the son/daughter is aware of the restrictions (*p* < .001, *d* = 1.1761) (Figure 6(b)).

### Impact of individual differences

**Figure 7.**
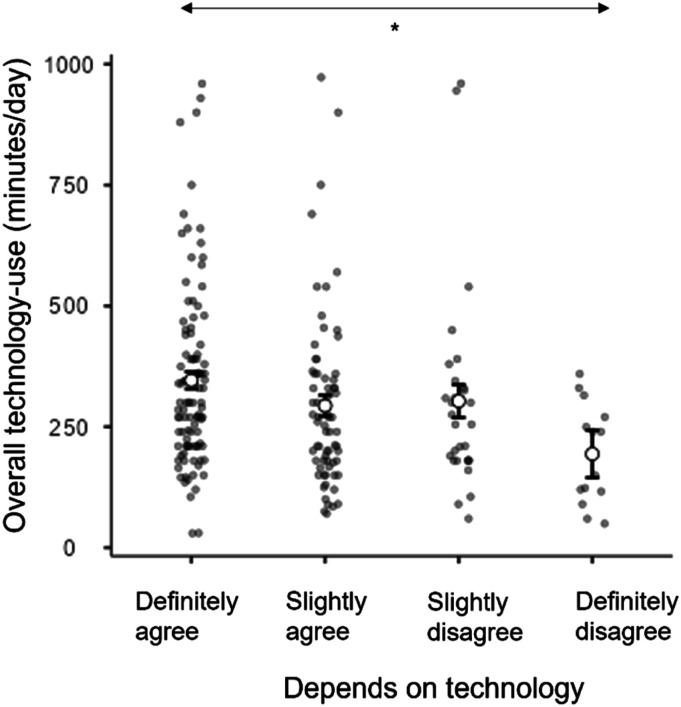
Estimated marginal means plots showing the differences in overall technology-use grouped by whether their son/daughter depends on technology. * *p* < .05, ** *p* < .01, *** *p* < .001.

Almost all participants agreed with the statement that technology was important to their son/daughter’s everyday life (98.2%). An ANOVA found that overall technology-use (DV) varied according to whether parents/caregivers reported their son/daughter depended on technology (IV; definitely agree, slightly agree, slightly disagree, definitely disagree), *F*(3,213) = 3.42, *p* = .018, η_p_^2^ = .046. Posthoc Tukey group comparisons revealed the only significant difference in technology-use was between those definitely agreeing and definitely disagreeing that their son/daughter depends on technology (Figure 7).

A paired-samples t-test comparing time spent on technological and non-technological activities revealed that individuals with DS spent longer on activities using technology (*M* = 309, *SD* = 181) than activities that don’t use technology (*M* = 184, *SD* = 135), *t*(204) = 7.82, *p* < .001, *d* = .546. Interestingly, this differed by age group, with no significant difference in children, *t*(89) = 0.86, *p* = .393, *d* = .091, BF_10_ = 0.166, but significant differences in adolescence, *t*(52) = 7.45, *p* < .001, *d* = 1.02, and adults ,*t*(61) = 6.54, *p* < .001, *d* = .830. This seems to be driven by a relatively longer time spent on technological activities in adolescence (*M* = 342, *SD* = 170) and adulthood (*M* = 418, *SD* = 215) relative to childhood (*M* = 214, *SD* = 94). Pearson’s correlations revealed that those who scored more highly on user-difficulty spent less time using technology, *r*(202) = -.206, *p* = .003. There was no significant association between user-difficulty and overall gaming-use, *r*(131) = -.087, *p* = .322, BF_10_ = 0.177 (moderate evidence for null hypothesis), nor overall social media-use, *r*(78) = -.201, *p* = .077, BF_10_ = 0.653 (anecdotal evidence for null hypothesis).

As individuals with DS in this study spanned 5 to 35 years old, it was important to understand how patterns of technology-use varied with age. A Pearson’s correlation revealed a significant positive association between age (months) and overall technology-use, *r*(220) = .478, *p* < .001, and between age and overall social media-use, *r*(85) = .272, *p* = .012, but no significant association between age and gaming, *r*(135) = .146, *p* = .092, BF_10_ = 0.440 (anecdotal evidence for null hypothesis). There was also a significant association between age and user-difficulty score, *r*(202) = -.266, *p* < .001, indicating that older participants tended to have fewer difficulties with using technology.

Partial correlations were carried out to examine whether associations between different types of technology-use were still evident after controlling for age. Overall technology-use had a positive association with gaming-use, *r*(135) = .417, *p* < .001, and to a lesser extent with social media, *r*(85) = .223, *p* = .042. After controlling for age, there was no association between gaming-use and social media-use (*p* = .412), indicating that the age-related patterns of use differed between the two activities. Interestingly, the association between social media-use and parental concerns relating to social media, was non-significant after accounting for age (*p* = .119), but the associations between social media concerns and a lack of parental information remained significant, *r*(88) = .371, *p* < .001. This suggests that the association between a lack of information and concerns were present regardless of the son/daughter’s age.

## Discussion

Access to technology provides individuals with opportunities to be creative, to build and maintain social contacts, to enjoy leisure activities such as gaming, to access educational activities, and to develop independence e.g., through assisted navigation (Livingstone & Bober, 2013). This study has enabled us to identify patterns of technology-use in people with DS. This will not only be of interest to parents of people with DS; it will also be useful for those seeking to make use of technology in interventions to support people with DS, and in understanding when technology might be a barrier for these individuals. This study had three main research aims; the findings relating to each aim are summarised below.

*Patterns of technology-use*: The most common devices used by people with DS were televisions, tablets, smart phones, and laptops, similar to the those identified previously in people with DS (Fritz, 2017), and the most popular devices used in the wider population identified in the Ofcom report (Ofcom, 2021). The range of activities for which technology is used was quite wide and included activities for fun and enjoyment, as well as activities that support learning and everyday living. This reflects previous findings with atypically developing groups where technology was used for leisure as well as for support (Ramsten et al., 2020), particularly for communicating with family and friends (Caton et al., 2022). The proportion of respondents from our sample who engaged with gaming was lower than in the wider population, but for those who did, the amount of time spent gaming per day was very similar (Ofcom, 2021). There was a much lower level of engagement with social media for people with DS, particularly in children, where only 12.1% of 5- to 12-year-olds used social media compared with 55% of typically-developing 5- to 15-year-olds (Ofcom, 2021). The lower use of social media in our sample was due primarily to a lack of interest and/or because they were not allowed to access social media. The most popular platforms used by this sample (WhatsApp and FaceTime) are primarily designed to communicate with friends and family, whereas the most popular platforms with typically developing young people (Instagram, Snapchat, and Facebook) are generally used to share information more widely (Ofcom, 2021). This preference for using technology to communicate with family and friends is consistent with a study of people with learning disabilities undertaken during the Covid-19 pandemic, where about half of the sample used technology for videocalls, but only 10% engaged with social media (Caton et al., 2022b). Overall, the current study shows that although the devices being used by people with DS are largely the same as typically developing individuals, the activities undertaken on the devices were not always similar.

Almost all respondents felt that technology played an important role in their son/daughter’s life, and gave them an opportunity to feel more involved. Despite this, almost three-quarters worried that their son/daughter used technology too much, which could suggest that some technology-based activities, such as gaming, are perceived as less ‘important’ than others. This is somewhat surprising given that games can motivate players to maintain engagement through the setting of goals, celebrating achievements, and encouraging perseverance (Granic et al., 2014). While it is currently unknown whether this translates to improved motivation in real-world situations, computer-based games offer a valuable opportunity for skill development. Responses indicated that visual and auditory features of technology-based activities were useful for maintaining their son/daughter’s attention for longer than non-technology activities. However, over half of participants had difficulties using a mouse, trackpad, keyboard, and voice-activated technology, and only a fifth could operate technology independently. This supports findings where challenges of using a keyboard or mouse were barriers to technology-use in individuals with intellectual disabilities who also struggle with fine motor skills (Kversøy et al., 2020). This suggests that there might be potential to improve access for people with DS still further through the use of adapted hardware, the use of touchscreen technology over a mouse or trackpad, and potentially through specific training in technology-use.

*Impact of parent/caregiver on technology-use*: Parents/caregivers were generally confident users of technology themselves, and felt able to support their son/daughter with their technology use. Parents/caregivers whose sons/daughters used social media largely felt that it was an important aspect of communication and friendship, however many felt they needed further information about social media, particularly the impact it might have and how other parents/caregivers monitor activity. Concerns such as the impact on their son/daughter’s mental health and to a lesser extent online bullying and accessing inappropriate material, are similar to concerns raised by caregivers of people with intellectual disabilities about internet access (Heitplatz et al., 2022), and to those identified in studies with parents of typically developing children and teenagers (Nikolopoulou, 2020; Sorbring, 2014).

The level of parent/caregiver engagement with gaming or social media, did not relate to the level of engagement in these activities in their son/daughter. However, there was a lower level of social media use where parents/caregivers had greater concerns about social media. This suggests that variation in the level of some types of technology-use may be due to parental concern rather than parental confidence in using technology or supporting their son/daughter. This contrasts with a study of typically developing teenagers, which found that parent concerns were related to their own confidence with using technology (Sorbring, 2014). In the study here, those parents/caregivers with greater concerns about social media, tended to impose greater time and access restrictions on their son/daughter, although interestingly, the level of gaming and social media use did not differ by the level of restriction.

*Impact of individual differences*: Those who reported greater difficulties with using technology also reported a lower level of technology-use, but user-difficulty had no impact on gaming or social media use specifically. It is possible that individuals generally played games which used features that they find most accessible and easy to use, such as touchscreen technology. In the case of social media, it is possible that the lack of impact of user-difficulty on level of use may be due to the age of participants; there was greater use of social media amongst older participants and user-difficulties reduced with age. It is not clear from this study why older participants had fewer difficulties, but it may be that they have developed strategies for overcoming difficulties, or selectively use technology so that they don’t encounter problems. These findings highlight the importance of designing technological activities and interventions that consider accessibility difficulties. This is particularly pertinent in view of the study which found greater gains made by participants with DS on a maths intervention using technology compared with a non-technological intervention (Ortega-Tudela & Gómez-Ariza, 2006). This highlights the potential for technology to be effectively used to develop skills in people with DS, but consideration should be given to the design of such interventions to ensure accessibility difficulties do not reduce their effectiveness.

### Implications

This study has identified important information for parents of individuals with DS and for those designing technology-based interventions and support programmes.

*Parents/Caregivers*: The patterns of use provided in this paper can be used as a reference for parents/caregivers uncertain about the extent to which they should monitor or limit their son/daughter’s activities, or the type of activities their son/daughter undertakes using technology. We also identified that the level of expertise varies amongst parents/caregivers, and that there is an interest in learning more, particularly about the impact of using social media. The findings may give parents/caregivers confidence to ask questions without worrying that they might be the only ones without answers. The findings also highlight a need for more parental information/resources regarding their child’s safe use of technology and social media.

*Designing technology-based activities*: An important outcome from this study is the extent to which people with DS have difficulties with using technology, particularly speech recognition, using a mouse, and limitations associated with cognitive skills such as reading. Technology and game designers should use these findings to better address the diverse needs of those with neurodivergent conditions, specifically people with DS to maximise the effectiveness of their products. It is also noteworthy that some parents felt there were no programmes available which would allow their son/daughter to gain greater independence such as life skills and road safety, and some parents were unaware of what tools/apps are available. For those parents/caregivers and individuals with DS who would like to make greater use of technology, an important step is ensuring there is easily-accessible parental information about what is available and how it can support individual with DS.

### Limitations and future work

Given the online nature of recruitment and data collection used in this study, those who participated may be more “tech-savvy” and the views of individuals with limited access to technology may not have been captured. However, this number is estimated to be very small, with 96% of households now having access to the internet (Ofcom, 2021). Additionally, the use of a parent-report questionnaire in this study leads to potential bias, and a risk of over- or under-representation of screen-time based on perceived social norms (Orben & Przybylski, 2019a; Scharkow, 2016). There may also be issues with the accuracy of reporting from parents’/caregivers’ of older or more independent sons and daughters, who may be less familiar with their son/daughter’s technology-use patterns. Using this study for guidance, future research could include more precise measures of technology-use, e.g., recording of actual screen time or technology-use diaries (Ellis et al., 2019). Alternatively, a more nuanced understanding of technology/social media use in people with DS would be revealed through interviews with people with Down Syndrome themselves or with their parents/caregivers*.* By contrast the use of a questionnaire-based design, allowed for extensive recruitment which led to this study being adequately powered. The sample size of this study compares very favourably to other studies with groups with neurodivergent conditions, which in turns leads to more reliable and robust findings. Finally, similar to other studies investigating the effects of technology-use, these findings are associational, and it is impossible to interpret cause and effect. Despite this, studies like this one are vital for laying the foundations for future intervention work, by identifying promising intervention targets.

### Conclusion

This study is an important first step into understanding how technology could be harnessed to improve support for people with DS, and for designing technology-based products to enable people with DS to have greater independence. Additionally, further research into the positive and negative impacts of technology-use in people with DS could offer reassurance to families concerned about their son/daughter’s use of technology.

## Supplemental Material

Supplemental Material - Examining the prevalence and type of technology-use in people with Down syndrome: Perspectives from parents and caregivers Supplemental Material for Examining the prevalence and type of technology-use in people with Down syndrome: Perspectives from parents and caregivers by Su Morris, Emily K. Farran, and Katie A. Gilligan-Lee in Journal of Intellectual Disabilities
