# Supplementary material for: Examining the prevalence and type of technology-use in people with Down syndrome: Perspectives from parents and caregivers
Source: J Intellect Disabil. 2023 May 18;28(3):683–705. doi: 10.1177/17446295231176121 (PMC11465601; doi:10.1177/17446295231176121)
Supplement: Supplemental Material - Examining the prevalence and type of technology-use in people with Down syndrome: Perspectives from parents and caregivers [file sj-pdf-1-jld-10.1177_17446295231176121.pdf]

Examining the prevalence and type of technology-use in people with Down syndrome: Perspectives from parents and caregivers Supplementary information and analyses

S2 Method

S2.1 Participants

Broad categories of additional diagnoses were created from participant responses to an open question. Two researchers grouped the diagnoses independently, and then discussed and agreed the final categories. There was 100% agreement between the researchers after the discussion. The broad categories of responses are reported in **Table 1** of the main manuscript.

**Motor**	**Health**	**Senses**	**Behavioural**
Dyspraxia	Heart	Visual impairment	ASD (Autism Spectrum Disorder)
Bone problems	Leukaemia	Hearing impairment	Tourette's
Hypermobility	Diabetes	Sensory processing disorder	Intellectual disability
Hypotonia	Thyroid	Skin	ADHD (Attention Deficit Hyperactivity Disorder)
	Epilepsy	Speech	Oppositional defiance disorder
	Asthma		Dyslexia
	Digestion problems		Social anxiety
	Coeliac		
	Brain problems		

S3 Results

S3.1 Patterns of technology-use across the sample

S3.1.1 Time spent on activities

Data were collected about estimated time spent on different devices and activities. It should be noted that it is difficult to measure time spent online accurately using a questionnaire measure, so these could be considered as estimates.

Across the sample, there was wide variation in the mean number of hours spent on different devices per day (**Figure s1**). The longest time was spent on tablets (1 hour 56 minutes) followed by watching TV (1 hour 40 minutes). Overall, the mean time spent per day on technological devices was 5 hours 13 minutes, while time spent on activities which don't use technology (such as performing arts, reading, and sports) was 3 hours 4 minutes.

Figure s1

Mean of time spent on each device per day across the sample.

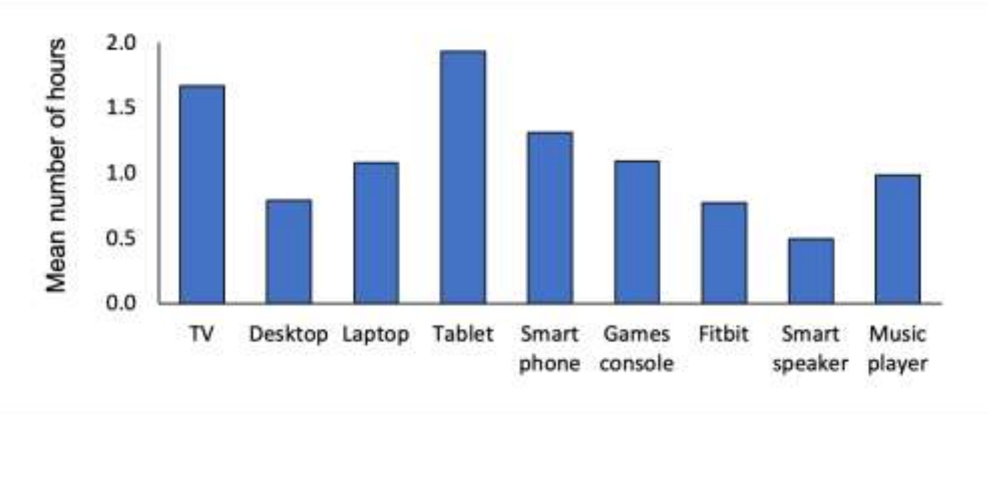

The mean time spent gaming per day across the sample was 1 hour 7 minutes. Individuals who used social media and gaming tended to access these activities on a daily basis (**Figure s2A**). The time spent on gaming varied according to the type of game being played. Generally, individuals played non-educational games such as character games or puzzle

games, for longer than educational games such as counting or reading games, with only 5.5% of individuals playing educational games for an hour or longer compared with 23.9% for non-educational gaming (**Figure s2B**). For social media, the mean time was very similar to gaming, at 1 hour 2 minutes. The majority of individuals who engaged with social media (76.8%), spent up to 30 minutes in a single session. The most frequently-given explanation for not engaging with gaming was that the individual was not interested (52.5%), whereas the main reason for never using social media was that they weren't allowed (44.5%), although a fairly large proportion also were not interested (32.4%) (**Figure s2C**).

Figure s2

Frequency and length of time spent on technology. ***A.*** *Frequency of access of gaming and social media activities.* ***B.*** *Length of time spent on different types of activities in a single session.* ***C.*** *Reasons why people with Down syndrome did not access gaming or social media.*

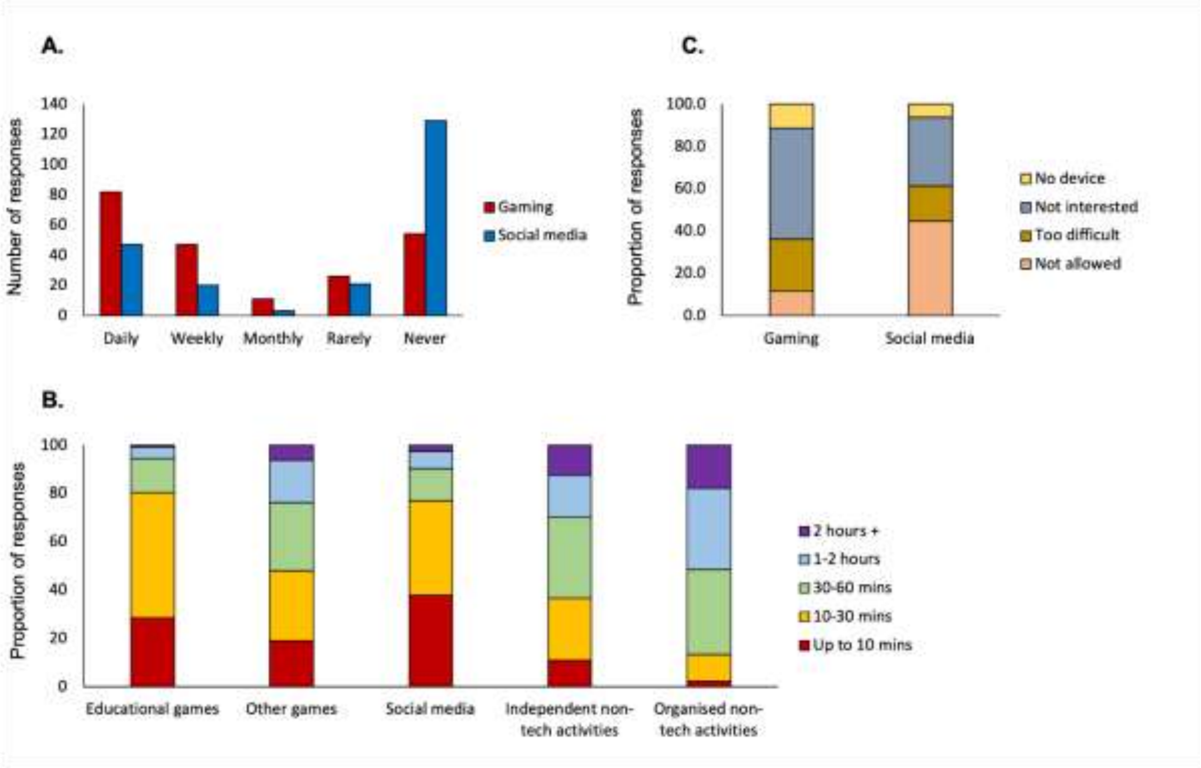

S3.1.2 Different types of technology, gaming, and social-media use

For those who engaged in gaming, most people with DS played computer games alone and almost all never played online with people they didn't know (**Figure s3**). 14.6% respondents sometimes or always played online with people they knew, while 74.8% sometimes or always played games with others in the same room.

Figure s3

The percentage of time gamers played alone or with others.

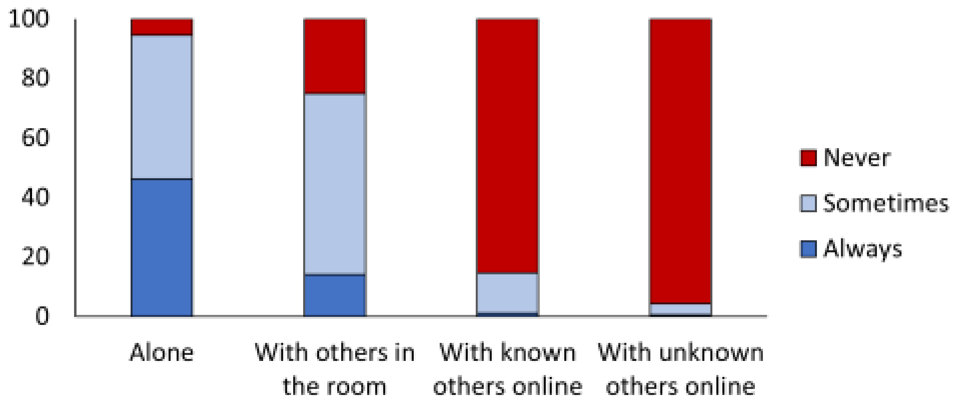

The participants with DS took part in a wide variety of non-technology activities (**Figure s4**). The most frequently given response was sporting activities, followed by meeting up with friends and taking part in performing arts.

Figure s4

Number of participants taking part in different non-technology activities

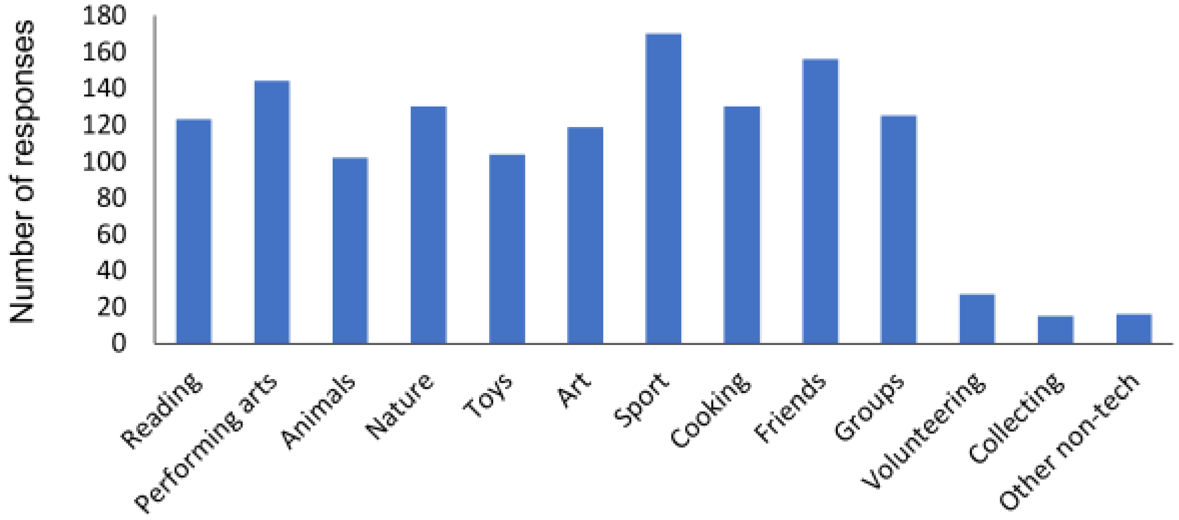

S3.1.4 Concerns and difficulties with technology-use

Most respondents (62.4%) definitely agreed that their son/daughter preferred using touchscreen over a mouse (**Figure s5A**). In most cases, respondents didn't specify either hardware or software adaptations (66.8%), and where adaptations were used, these were mostly software-related (26.2%) (**Figure s5B**). Hardware adaptations mostly related to the type of keyboard, including a font overlay and a keyboard for use on a lap, which formed part of the 'other' category (**Figure s5C**). Software adaptations were most frequently related to supporting reading, writing, and communication (**Figure s5D**).

Figure s5

Responses to questions about technology adaptations. ***A.*** *Proportion of respondents who prefer a touchscreen over a mouse.* ***B.*** *Proportion of respondents who require adaptations.* ***C.*** *Types of hardware adaptations.* ***D.*** *Types of software adaptations.*

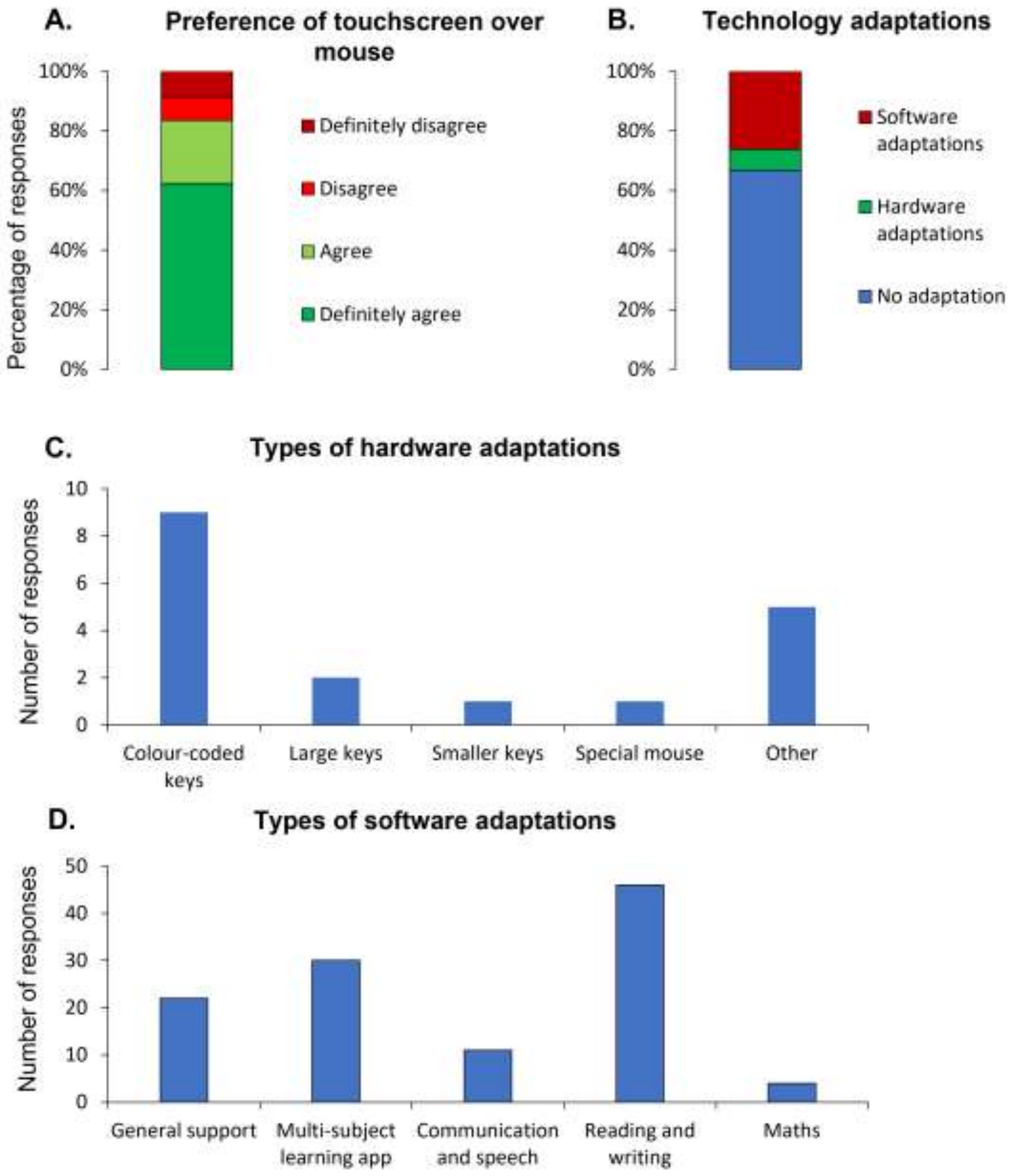

There were wide-ranging responses to the open-ended question about any additional barriers participants encounter, including difficulties with devices, parent/caregiver concerns, and limitations due to problems associated with DS. The most frequently-given response (20 respondents) was that their son/daughter's difficulties with

speech limited their technology-use, while 13 responses mentioned reading difficulties and 8 responses mentioned motor difficulties. Thirteen responses mentioned parent/caregiver concerns about their son/daughter's vulnerability and lack of understanding about the dangers of being online, and nine highlighted a lack of confidence or expertise on the part of the parent/caregiver. There were 19 comments about the high cost of hardware and software, and about problems with using the technology they already had, such as old devices and poor internet connection. Other limitations included a lack of time, lack of training for their son/daughter, and a lack of suitable apps available that are useful for real-world situations.

S3.2 Associations between parental characteristics and son/daughter's technology-use

In response to the question about time restrictions set by parents/caregivers, there were a few differences between the contexts of gaming and social media (**Figure s6A**). Similar proportions of respondents set time restrictions for gaming and social media, but there was a higher proportion of respondents who did not set any time restrictions for social media (45.1%) than gaming (26.1%). This may reflect the relative importance placed on both these activities by parents/caregivers, where gaming may be considered simply a fun past-time, while social media may be perceived as a way to develop social skills.

In terms of access restrictions for social media sites (**Figure s6B**), there was a similar proportion of parents/caregivers who restricted access via the device (38.6%), and whose son/daughter was aware of sites they were not permitted to access (36.1%). A lower proportion of respondents had no access restrictions to social media (25.3%) than had no time restrictions (45.1%), indicating that parents/caregivers had greater concern about which sites their son/daughter could access rather than the length of time they visited those sites.

Figure s6

Restrictions for people with Down syndrome on the use of gaming and social media. ***A.*** *Parent/caregiver time restrictions.* ***B.*** *Parent/caregiver access restrictions.*

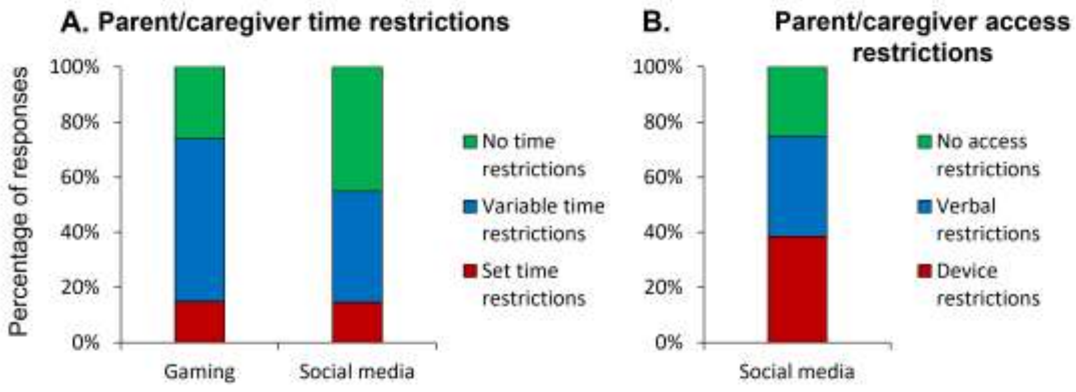

Three multivariate analysis of variances (MANOVAs) were completed to assess whether the level of time restrictions on first, gaming (IV1), and second, social media (IV2), and access restrictions on social media (IV3), could be explained by parental confidence scores in both technology-use (DV1) and giving support (DV2). There was no significant difference in parental confidence between type of time restrictions set on gaming (p = .192) or social media (p = .703), nor on access restrictions on social media (p = .232).

S3.3 Impact of individual differences

Difficulties with using technology

A series of ANOVAs were carried out to determine whether technology-use (DV1), gaming-use (DV2), or social media-use (DV3) varied according to the participants' quality of vision (IV: Better than average, average, poor, very poor). There were no significant differences due to vision (technology-use: p = .637, η_p^2 = .008; gaming-use: p = .738, η_p^2 = .010; social media-use: p = .264, η_p^2 = .048). A further ANOVA revealed that there was no significant difference (p = .693, η_p^2 = .007) in user-difficulties (DV) by quality of vision (IV). This perhaps reflects the emphasis on motor difficulties in the user-difficulty scale, rather than visual difficulties. Further, Pearson's correlations revealed that the number of additional diagnoses did not significantly associate with user-difficulties (p = .013), which is

perhaps unsurprising as many of the additional diagnoses would not necessarily have an impact on technology-use.
